# Best interests, benefit, will and preference: The influence of international human rights and external actors on decision-making frameworks in the United Kingdom and Ireland

**DOI:** 10.1016/j.ijlp.2022.101841

**Published:** 2022

**Authors:** Aoife M. Finnerty, Judy Laing

**Affiliations:** School of Law, University of Bristol, Bristol BS8 1RJ, United Kingdom

**Keywords:** Substitute decision-making, Legislative process, Best interests, CRPD, Advocacy groups, Will and preferences

## Abstract

This article examines the development of the law relating to decision-making on behalf of adults that lack capacity in Scotland, Ireland and Northern Ireland. Using two points of tension evident in the recent Northern Irish legislative consultation process, namely the suitability of the ‘best interests’ standard and international debate regarding domestic compliance with the United Nations Convention on the Rights of Persons with Disabilities (United Nations, 2006) this contribution exposes the impact of international human rights and influence of external actors, principally advocacy groups and their individual members, on the resulting legislation. Given the recency of the Irish and Northern Irish legislation and the current law reform review in Scotland, the article concludes by highlighting points of tension that may emerge between domestic standards for decision-making and the CRPD.

## 1 Introduction

Debates persist about who or what body should make decisions on behalf of adults that lack decision-making capacity and normative questions have arisen in relation to the appropriate standard for such decision-making. Should it be substituted judgement^1^ or one informed by best interests or welfare considerations? (Cantor, 1985, Cantor, 2001; Donnelly, 2016; English, 1993). More recently, since the advent of the United Nations Convention on the Rights of Persons with Disabilities (CRPD), debates have centred on whether there should be a system for making decisions on behalf of others at all (McSherry and Weller, 2010). While the legal frameworks on assisted decision-making in the common law jurisdictions in the United Kingdom (UK) – England & Wales, Scotland, Northern Ireland – and Ireland share a number of similar features, there are marked differences in the legal standard applicable to decision-making on behalf of adults lacking capacity.^2^ Broadly speaking, England & Wales and Northern Ireland occupy ‘one camp’ by utilising the ‘best interests’ standard, with Scotland and Ireland occupying the other, as Scotland currently utilises ‘benefit’ and Ireland uses ‘will and preferences’ with additional conditions, such as the requirement *inter alia* that any intervention must benefit the individual. This article looks beyond the legislation in Scotland, Ireland and Northern Ireland to discuss the processes behind the jurisdictions legislating in the manner they did.

Given that both Ireland and Northern Ireland legislated quite recently in this area and Scotland is currently considering reform, the divergence in approaches is noteworthy. However, more so are the factors that may have led to this divergence. Two apparent and linked points of tension were evident during the consultation phase of the Northern Irish legislative process – namely, views on ‘best interests’ and disagreement regarding the interpretation of the CRPD by the UN Committee on the Rights of Persons with Disabilities (the CRPD Committee – the body charged with monitoring treaty implementation). It is what these particular areas of tension can tell us about the influence of international human rights on domestic legislation and the impact of external actors, in particular civil society and advocacy groups, on the law-making process that is the focus of this article. Mental capacity law is manifestly an area where the human rights of the individual are engaged; decisions regarding medical treatment are intensely personal and frequently engage interests such as those in bodily integrity, privacy and self-determination. Consequently, the degree to which these domestic rules on decision-making reflect international human rights standards and related best practice is important; arguably, the more aligned legislation is with international human rights instruments and reflective of best practice, the less scope there is for the rights of the individual to be compromised.

The impact that advocacy organisations, interest groups, academics and those with lived experience can have on domestic legislation has emerged relatively recently as an aspect of law reform. Whilst there has always been a certain amount of consultation with the public and interested parties in shaping mental capacity legislation (Scottish Law Commission, 1995), increasingly more power and influence is being exerted on this area by civil society groups, advocacy groups and academics. For example, it has been stated by Flynn that civil society groups in Ireland were directly responsible for the change in name of the Irish Act (Flynn, 2021a). At consultation stage, it was referred to as the Mental Capacity Bill, however when the Bill was presented to the Irish Parliament, it had been renamed the Assisted Decision-Making (Capacity) Bill. This article highlights some of the voices that contributed to each legislative process and emphasises the impact these groups and individuals appear to have had on shaping domestic legislation. Such impact is also evident internationally, for example by the inclusive consultation process that led to the creation and drafting of the CRPD (Schulze, 2010: 83-89).

Our analysis partly draws on an approach to comparative legal research which is described as the functional method (Ralf, 2006). The functional approach starts from the basic assumption that: ‘the legal system of every society faces essentially the same problems, and solves these problems by quite different means though very often with similar results’ (Zweigert and Kötz, 1998: 102). This is regarded as the traditional method for comparative legal research, though it has been subject to critique for paying insufficient attention to the wider social context (Nelken, 2007: 3-42). We start with the assumption that each legal system shares a concern to address the same social problem i.e. the legal regulation of assisted decision-making for vulnerable persons. We do, however, take account of some of the wider features of the law reform process, for example, the way in which different groups and individuals help to shape the law. In documenting legislative committee debates, minutes of evidence and submissions throughout those reform processes, we use a ‘hidden lawmakers’ lens (Montgomery, Jones, and Biggs, 2014) to form some conclusions about the influence of external actors on the divergent approaches to law in this area. Unlike the work of Montgomery et al, however, we do not consider the legitimacy of such hidden ‘law-making’ processes.

We first set out relevant human rights instruments that impact, or ought to impact, on how all four jurisdictions approach this area of law, before outlining the relevant domestic legal frameworks: namely the Mental Capacity Act 2005 (MCA) in England & Wales; the Adults with Incapacity Act 2000 in Scotland (AWIA); the Assisted Decision-Making (Capacity) Act 2015 in Ireland (ADM(C)A); and the Mental Capacity Act (Northern Ireland) 2016 (MCA(NI)). In our analysis of the processes that ultimately led to the jurisdictions either adopting or rejecting the ‘best interest’ standard, the impact of international human rights law and influence of particular interest groups and individuals in informing the legal standard in each jurisdiction is explored. Finally, the article concludes by bringing together some overarching observations about the way in which the law has been shaped, and continues to be shaped, in this area.

## 2 The human rights context

This section exposes some tensions between existing international human rights frameworks, which are important to note from the outset, as they emerged in legislative debates and appeared to influence the way in which some jurisdictions legislated.

Regional and international human rights systems can provide important frameworks and norms for national laws on assisted decision-making. Notably, the enactment of the Human Rights Act 1998 (HRA) means that England & Wales, Scotland and Northern Ireland are directly bound by the European Convention on Human Rights (ECHR). The ECHR also became part of Irish law in 2003 when the European Convention on Human Rights Act 2003 was passed. The ECHR is a regional human rights treaty which guarantees a range of civil and political rights. Of particular relevance in this context are Article 2, which protects the right to life; Article 8, which protects the right to a private and family life and promotes the exercise of individual autonomy; Article 5, which protects the right to liberty; and Article 14, which enshrines the prevention of discrimination in the enjoyment of all ECHR rights. Legal regimes for assisted decision-making must be compatible with the ECHR with clear standards and processes in place to safeguard individual autonomy, prevent discrimination and guard against arbitrary deprivations of liberty. Legal regimes must also ensure that the right to life in Article 2 is protected and balanced against the exercise of Article 8. The necessity of correctly balancing these two rights can be brought into sharp focus in the context of decision-making on behalf of those who lack capacity.^3^

### 2.1 The UNCRPD

International human rights protection for persons with mental disabilities has been reinforced by the CRPD, which has generated debate about domestic laws and frameworks for assisted decision-making (Bartlett, 2012; McSherry and Weller, 2010; Stein, Mahomed, Patel, and Sunkel, 2021). The treaty was adopted by the UN General Assembly in 2006, ratified by the UK in 2008 and by Ireland in 2018. The CRPD is viewed by some as heralding a new vision of equality for persons with long-term mental and physical disabilities; for example, Kelly argues that while the CRPD does not have the status of ‘hard law’,^4^ it may increase pressure for countries ‘to address issues such as competence, capacity and consent amongst individuals with disabilities’ (Kelly, 2011: 451) and ‘still appear[s] to articulate a positive obligation to protect rights that is similar in magnitude’ to the HRA (2017: 96). The CRPD represents a ‘paradigm shift’ in international disability law (Bartlett, 2012; Richardson, 2012); non-discrimination is a core value and a wide range of social and economic rights are protected, in addition to civil and political rights. Many of the rights in the CRPD are already protected by other instruments, however it has been argued that the CRPD is special because it frames those rights in a manner which is specific for people with disabilities, who are rarely referenced in those other instruments (Szmukler, Daw, and Callard, 2014: 245; Kelly, 2017: 94). Article 12 specifically, guarantees the right to equal recognition before the law and for persons with disabilities to enjoy legal capacity on an equal basis. Thus, it is of particular relevance here as it may have implications for both well established and newer domestic mental capacity laws (de Bhailís and Flynn, 2017).

#### 2.1.1 General comment no. 1

In 2014, the CRPD Committee issued its first General Comment (United Nations Committee on the Rights of Persons with Disabilities, 2014). In it, the CRPD Committee acknowledged that ‘[a]ll persons with disabilities, including those with physical, mental, intellectual or sensory impairments, can be affected by denial of legal capacity and substitute decision-making’ (Committee, GC No 1, 2014, para. 9) and made a number of statements regarding how compliance with Article 12 should be achieved by signatories. The CRPD Committee interpreted Article 12 to mean that ‘perceived or actual deficits in mental capacity must not be used as justification for denying legal capacity’ and reaffirmed that:

[A] person’s status as a person with a disability or the existence of an impairment (..) must never be grounds for denying legal capacity or any of the rights provided for in article 12. All practices that in purpose or effect violate article 12 must be abolished in order to ensure that full legal capacity is restored to persons with disabilities on an equal basis with others (Committee, GC No 1, 2014, para 9).

Critically, the CRPD Committee stated that ‘support in the exercise of legal capacity (…) should never amount to substitute decision-making’ and that states were obliged to ‘review the laws allowing for guardianship and trusteeship, and (…) to develop laws and policies to replace regimes of substitute decision-making by supported decision-making, which respects the person’s autonomy, will and preferences’ (Committee, GC No 1, 2014, paras. 17, 26). Prior to outlining some criticisms of this interpretation, it is worth stating that there are undoubtedly those who support states pivoting to a legal system of supported decision-making and the need to abolish any form of substitute decision-making (Flynn and Arstein-Kerslake, 2014; Minkowitz, 2010).

#### 2.1.2 Critiques of the general comment

The CRPD Committee’s interpretation of Article 12 undoubtedly creates challenges for national systems as it effectively outlaws all forms of substitute decision-making regimes based on mental (in)capacity (Dawson, 2015; Hoffman et al., 2016). Szmukler argues that the ‘absolutist position of the CRPD Committee’ is so ‘dramatically at odds with centuries of legal acceptance of involuntary detention and treatment’ that it is no surprise that it has received harsh criticism (Szmukler, 2019). The critiques have originated from many sources – medical professionals, academics, legal practitioners – and stem from a range of practical and theoretical issues. We set out below a brief overview of some of the primary criticisms as it provides some useful wider context to frame the legislative debates in each jurisdiction. Though the critiques have been loosely grouped below, it is worth noting they are often related to one another.

Broadly speaking, the first tranche of criticisms relate to ‘issues of consistency’ in the sense that the interpretation of Article 12 is inconsistent with other international treaties and instruments, established legal principles and the CRPD itself. Dawson, for example, believes that challenges for domestic implementation of the CRPD are compounded by ambiguities and inconsistencies between different rights in the CRPD (2015). He also argues that aspects of the CRPD Committee’s interpretation of Article 12 do not seem compatible with ‘sophisticated legal systems’ and basic legal principles such as intent, knowledge, foresight and the ability to process information (2015: 73). As a result, it has been argued that full compliance with the CRPD as interpreted by the CRPD Committee is ‘quite difficult and potentially impossible’ (Hoffman, Sritharan and Tejpar, 2016: 4; see also Davidson et al., 2016: 32). Freeman et al. (2015) argue that the CRPD Committee’s interpretation ‘threatens to undermine critical rights for persons with mental disabilities, including the enjoyment of the highest attainable standard of health, access to justice, the right to liberty, and the right to life’ (2015: 844).

Some commentators argue that the interpretation is in conflict with the ECHR, as well as many domestic laws which allow for assisted decision-making regimes on the basis of mental (in)capacity (Fennell and Khaliq, 2011; Martin et al., 2014). Indeed, Szmukler argues that the European Court of Human Rights’ interpretation of Article 12 does not appear to concur with that of the CRPD Committee given the judgment in *AM-V v Finland*^5^ (2019), wherein it was concluded that the ‘balance between the respect for the dignity and self-determination of the individual and the need to protect the individual and safeguard his or her interests’ was properly struck despite the decision being contrary to the will and preferences of the individual (*AM-V v Finland*, para. 90). It has also been noted that the CRPD Committee’s view is not shared by other UN bodies, for example, the UN Subcommittee on Prevention of Torture (UN Subcommittee, 2016).

A second tranche of objections relate to whether the CRPD Committee’s interpretation is correct based on the wording of the CRPD itself. The Report submitted to the UK Ministry of Justice by the Essex Autonomy Project (EAP) was unambiguous in its view, stating that CRPD Committee ‘is not correct in its claim that compliance with the CRPD requires the abolition of substitute decision making and the best-interests decision-making framework’ (Martin et al., 2014: Executive Summary). The authors contend this for a number of reasons, starting with the argument that the claims made by the CRPD Committee ‘go beyond anything that is explicitly stated in the text of the CRPD’ and that the CRPD does not ‘actually say that substitute decision-making should be abolished’ nor does it ‘state that the best-interests paradigm must be replaced’ (Martin et al., 2014: 13). It is worth noting, however, that EAP does not suggest that the way in which best interests is assessed under the MCA is compliant with the CRPD.

The authors also raise questions regarding the very meaning of the word ‘respect’ in the context of Article 12, suggesting that the CRPD Committee’s interpretation is ‘predicated on an understanding of “respect” that is not the primary dictionary definition of the term, is implausibly stringent in the present context, and would lead to incoherent policy injunctions’ and related to the first category of criticisms, that it would lead to conflict of rights within the CRPD (Martin et al., 2014: 43-4). They also highlight that the CRPD wording is ‘respect the *rights*, will and preferences of the person’– not merely their ‘will and preferences’ – and this confers an obligation on states to protect the human rights of the individual (Martin et al., 2014: 42-4).

Finally, commentators object to the CRPD Committee’s interpretation on the basis of the harm that may come to persons with disabilities if that interpretation is realised in domestic legislation. Such critiques often acknowledge ‘the impediments to agency’ of the individual (Donnelly, 2016: 324), arguing that the presence of protection or safeguards in capacity legislation is necessary for those lacking capacity and highlighting the existence of situations in which decisions ought to be taken that do not accord with (either) the will or preferences of the person. Scholten and Gather, for example, outline six adverse consequences of the CRPD Committee’s interpretation for individuals with mental disability, including an adverse effect on the well-being and autonomy of the person (Scholten and Gather, 2018). They also comment that it may aggravate ‘the problem of undue influence’, which Ward also notes:

As soon as others play a supportive role for a person with some degree of intellectual disability, questions may arise in some cases as to whether to some extent we are hearing the voice of the supporter rather than that of the disabled person, whether undue influence is being exercised, or whether what is in fact occurring is unregulated substitute decision-making (Ward, 2014).

In relation to support, Donnelly cautions that when one considers the range of people who will require support to make a decision as a result of impaired capacity, there will be situations in which that support will simply be inadequate to make an autonomous decision (2016: 324).

The analysis above sought to highlight two important aspects; first is the degree to which there is significant debate regarding the correctness of the CRPD Committee’s views on Article 12. The second is the degree to which the desirability of such an interpretation is contested (assuming that it is ‘correct’ in the first place). As will be discussed below, this significant disagreement has affected how some jurisdictions have drafted mental capacity legislation. Accordingly, it is worth considering these jurisdictions and their related approaches to law reform as occupying a place on a continuum rather than viewing compliance (or not) with the CRPD as absolute.

## 3 The domestic context

Though best interests is closely associated with the MCA, it had been the standard at common law for decision-making on behalf of adults lacking capacity in England & Wales since the late 1980s. As a result, the law in England & Wales operated as a sort of benchmark when the legislative processes were taking place in Scotland, Ireland and Northern Ireland and is briefly set out below.

### 3.1 England & Wales

The decision in *Re F*^6^ established that an individual that lacked decision-making capacity could be treated in her ‘best interests’. However, best interests was to be determined by reference to the *Bolam*^7^ test, which considers if a medical professional has acted in accordance with generally accepted practice. As a result, best interests was to be a judgement by the medical professional. Over the past three decades, best interests has migrated somewhat from initially being almost exclusively concerned with a person’s medical interests – as was the case at common law in *Re F* – to incorporating factors outside of what may be clinically indicated, such as ‘emotional, psychological and social benefit’,^8^ ‘patient views,’^9^ the ‘past and present wishes and feelings of patient’ and ‘the factors which he would consider if able to do so’.^10^ Section 1(5) of the MCA codified the common law position and expanded the definition of best interests beyond exclusively medical or clinical considerations.

Section 4 of the MCA sets out the relevant considerations when a decision is being made on behalf of a person lacking capacity. Insofar as is practical, the person who lacks capacity (‘P’) should be permitted, encouraged and assisted to participate as fully as possible in decisions relating to them (MCA, s. 4(4)). Of key importance is what has become known as ‘the checklist’ contained in section 4(6) for determining P’s best interests, which states that the decision-maker should consider, insofar as they are reasonably ascertainable:

(a) the person’s past and present wishes and feelings (and, in particular, any relevant written statement made by him when he had capacity),

(b) the beliefs and values that would be likely to influence his decision if he had capacity, and

(c) the other factors that he would be likely to consider if he were able to do so.

Section 4(7) of the MCA identifies a number of parties who should be consulted as to the best interests of P, provided that it is appropriate and practicable to do so; some examples include those identified by P as persons to be consulted (MCA, s. 4(7)(a)) and those involved in P’s care or interested in his welfare (MCA, s. 4(7)(b)). Consequently, it can be argued that the interpretation of best interests in the MCA by the courts, at least in the last decade, has generally been wider and more person-centred than the interpretation contained at common law in *Re F.*^11^ It is important to bear in mind this shift from medically focused best interests to a more holistic interpretation in the discussion of the consultation processes in Scotland and Ireland below. Despite the former occurring almost 20 years before the latter, very similar arguments about the relationship between paternalism and best interests featured in both processes.

### 3.2 Scotland

In contrast to its counterparts in the rest of the United Kingdom and Ireland, the Scottish legislative framework in this area has been established for some time. Indeed, the Scottish framework pre-dated the MCA and was referred to multiple times when the MCA consultation was taking place in the early 2000s (Joint Committee on the Draft Mental Incapacity Bill, 2002-2003). Amongst the guiding principles in the AWIA for intervention in respect of a person that lacks decision-making capacity is that of ‘benefit’, in contrast to the MCA concept of best interests. Section 1(2) of the AWIA states:

There shall be no intervention in the affairs of an adult unless the person responsible for authorising or effecting the intervention is satisfied that the intervention will benefit the adult and that such benefit cannot reasonably be achieved without the intervention.

Consequently, unless the intervener can demonstrate that the act will likely benefit the person deemed to lack capacity and that said benefit cannot be achieved without intervention, the intervention will not be appropriate in the circumstances. Furthermore, as is common across the jurisdictions to promote autonomy and liberty, the intervention must be the least restrictive option (AWIA, s. 1(3)).

In determining both whether an intervention should be made and what that intervention should be, the intervener is required to take account of the past and present wishes and feelings of the individual, insofar as they are ascertainable, in addition to the views of a number of parties, such as the nearest relative, insofar as ‘it is reasonable and practicable to do so’ (AWIA, s. 1(4)). Specifically in relation to medical treatment, Part 5 of the Act permits specified medical professionals to certify that an individual lacks capacity and to make medical decisions on their behalf in order to safeguard or promote their physical or mental health subject to certain conditions (AWIA, s. 47). In line with the guiding principles, medical professionals should provide treatment if it is the least restrictive method of benefitting the person in line with their reasonably ascertainable prior or current wishes and feelings, following consultation with the relevant parties, if appropriate (Code of Practice for Medical Practitioners, 2010: para. 1).

### 3.3 Ireland

In contrast to Scotland, the law in Ireland has been the subject of a very recent and heavily protracted reform process, aspects of which are explored in more detail below. After considerable consultation, the Irish legislature settled on the inclusion of ‘will and preferences’ – as opposed to best interests – amongst the guiding principles in the ADM(C)A. However, ‘will and preferences’ does not stand alone and amongst the guiding principles is that of ‘benefit’; in other words, the intervener should act in good faith at all times and for the benefit of the individual (ADM(C)A, s. 8(7)(e)). Under Irish law the person intervening in respect of an individual shall give effect to the past and present will and preferences of the individual, provided that they can reasonably be ascertained (ADM(C)A, s. 8(7)(b)) and intervene in a manner that minimises the restriction of the person’s rights and freedom of action (ADM(C)A, s. 8(6)(a)). The intervention must also be made with due regard to the need to respect the right of the relevant person to dignity, bodily integrity, privacy and autonomy (ADM(C)A, s. 8(6)(b)).

It is worth stating that while the ADM(C)A was enacted in 2015, full commencement was a slow process with several delays. At the time of writing, full commencement is expected to take place in November 2022 (Decision Support Service, 2022). This is because the passing by both houses of the Irish Parliament of the Assisted Decision-Making (Capacity) (Amendment) Bill 2022, which amongst other functions will enable the Decision Support Services to carry out its role under the Act, will not take place until that time.

### 3.4 Northern Ireland

Within the last decade, legal changes were also afoot in Northern Ireland to address a lack of capacity legislation and amend mental health legislation. After a considerable period of consultation which began in the early 2000s, the MCA(NI) received Royal Assent in May 2016. Similar to its counterpart in Ireland, however, the MCA(NI) is also only partially commenced. At this stage, it is not clear when it will be fully commenced, but the provisions relating to healthcare decisions on behalf of persons who lack capacity are not yet in operation.

Similar to the MCA, an act done or a decision made for or on behalf of a person who lacks capacity should be done in the person’s ‘best interests’ (MCA(NI), s. 2) and in a way that is minimally restrictive to the rights and freedom of that person (MCA(NI, s. 7(8)). In determining the best interests of the individual, the MCA(NI) sets out a similar checklist to the one found in s. 4(6) of the MCA. Insofar as they are reasonably ascertainable, the decision-maker must have ‘special regard’ to P’s past and present wishes and feelings, their beliefs and values and the other factors that P would likely consider if able to do so (MCA(NI) s. 7(6)). Although the wording is somewhat stronger than the corresponding section of the MCA, it remains to be seen if there will be a significant difference in practice between how ‘special regard to’ and ‘consider’ are interpreted by the courts, particularly given the caveat of reasonable ascertainability. In any event, in contrast to Scotland and Ireland, it is the best interests of the individual that must guide decisions made on their behalf in Northern Ireland.

## 4 Discussion

Having briefly laid out the relevant domestic frameworks, attention now turns to the impetus behind each jurisdiction legislating in the way that it did. As noted above and further explored below, the first theme that emerged from our analysis of the various legislative consultations was the role played by international human rights in domestic law-making discourse and processes. CRPD compliance appears to have considerable importance in Ireland and Scotland, and can also be seen, albeit a different manifestation, in the approach taken in Northern Ireland, where clear concerns were expressed regarding the consistency of aspects of the CRPD with national obligations under the ECHR.

The second theme that emerged, in particular from the legislative committee debates and reports, was the varying influence that external actors had on shaping the law and the degree to which such influence can have a strategic element. In our view, the Irish consultation process that preceded the passing of the ADM(C)A exemplifies both the former and the latter. The variable levels of influence that such groups can have on the direction of legislation is also highlighted when one considers the current Scottish reform consultation and the legislative process in Northern Ireland. These two themes will be considered in turn below, but it is worth saying at the outset that they are somewhat linked; throughout the legislative processes, those submitting to the various parliamentary committees can be seen to use human rights arguments and norms in their written submissions and oral evidence to influence legal change.

### 4.1 ‘Best interests’ and the influence of international human rights in Scotland and Ireland

Within all jurisdictions, best interests has been a polarising concept to say the least. The AWIA, which predated the UK’s ratification of the CRPD, was based on recommendations contained in the Scottish Law Commission ‘Report on Incapable Adults’ dating back to 1995. The aim of that Commission was to devise principles that ‘empower[ed] adults with incapacity, respecting their rights and recognising that many have been managing their own affairs in the past’ and that some would ‘be able to do so again in the future’ (Scottish Law Commission, 2012: para 3.7). The view of the Commission was that best interests failed to ‘give due weight to the views of the adult, particularly to wishes and feelings which they had expressed while capable of doing so’ and was ‘too vague’ by itself (Scottish Law Commission, 1995: para. 2.50). Critically, the Commission stated:

Incapable adults such as those who are mentally ill, head injured or suffering from dementia at a time when a decision has to be made in connection with them, will have possessed full mental powers before their present incapacity. We think it is wrong to equate such adults with children and for that reason would avoid extending child law concepts [i.e. best interests] to them. (Scottish Law Commission, 1995: para. 2.50)

Arguably, this was a legitimate concern in relation to best interests given that case law in England & Wales at that time, for example, *Re F* and *Re MB (An Adult: Medical Treatment)*^12^, explicitly acknowledged the commonalities between child welfare law and decision-making for those lacking capacity.^13^ However, that is not to say that even with ‘the more expansive, arguably revolutionary, view of best interests under the MCA’ (Donnelly, 2016: 33), the current MCA legal position is not open to criticism for a variety of reasons, including failing to give ‘sufficient certainty (…) on how much emphasis should be given to the person’s wishes and feelings’ (Law Commission, 2017) and for failing to give consistent and sufficient weight to the wishes and feelings of the person (Donnelly, 2016: 330). As such, there have been arguments in favour of modifying s. 4 MCA and how best interests is determined (Donnelly, 2016; Martin et al., 2014; Ruck Keene and Auckland, 2015).

More recently, the conversation in Scotland has also turned to law reform with the Executive Team of the Scottish Mental Health and Incapacity Law Review (Scottish Mental Health Law Review Executive Team, n.d) publishing its proposals for reform of Scottish mental health and incapacity law in 2022. There appears, at the very least, to be no appetite for a move towards best interests decision-making for adults that lack capacity. Rather, where aspects of Scottish law could be construed as ‘suggestive of a paternalistic best interests approach’, the Scott Review has advocated for a position that maximises ‘the autonomy of the adult and respect[s] their will and preferences’ (Scottish Mental Health Law Review, 2022a: 164). For example, on section 47 of the AWIA, which permits a medical professional to do ‘what is reasonable’ in order to safeguard or promote a person’s physical or mental health once a certificate of incapacity has been issued, the Scott Review has proposed reframing the authority granted by the section ‘to make clear that treatment which is authorised should be that which would reflect the best interpretation of the adult’s will and preferences’ (Scottish Mental Health Law Review, 2022a: 169; Scottish Mental Health Law Review, 2022b: 716). This seems to indicate that Scotland may move its incapacity law in a more CRPD-focused direction. Indeed, all reports published as part of the Scott Review evidence the degree to which the CRPD is at the forefront of current debates in Scotland.^14^ The relevance of the CRPD to the work of the Scott Review predates the start of the review process, dating at least as far back as the Scottish government’s delivery plan for the CRPD – ‘A Fairer Scotland for Disabled People’ (Scottish Government, 2016: 14-15). This evidences explicit commitment from the Scottish government to recognise and implement the CRPD, which should certainly be considered in light of the CRPD Committee’s investigation into the UK’s compliance with the CRPD in 2016 (UN Committee on the Rights of Persons with Disabilities, 2016).^15^ That is not to say, however, that there is agreement with the CRPD Committee’s interpretation of Article 12 in Scotland, as will be explored further below.

A reoccurring theme in the current Scottish reform process is the importance of human rights; indeed, amongst the principles underpinning the Scott Review consultation process is respect for core human rights, with the Final Report stating that ‘[h]uman rights are critical to our recommendations’ (2022b: 23). Accordingly, many of the amendments that have been suggested by the Scott Review have been framed or expressed in terms of human rights. For example, it has been recommended that duties to provide health and social care ‘should be reframed in terms of human rights standards’ and that budgeting decisions should be made in a way that ‘reflects human rights principles’ (2022a: 37-41). More broadly, Scotland’s desire to be a world leader in human rights appears to be driving recent reforms in multiple areas of law and policy (National Taskforce for Human Rights Leadership, 2021: 6). Arguably, this agenda has been furthered by the work of the Scott Review and recent incorporation of the United Nations Convention on the Child directly into Scottish law. Not only has the National Taskforce for Human Rights Leadership in Scotland called for the direct incorporation of rights from pre-existing instruments, but also recommended that rights not currently protected by UN treaties – such as the right to a healthy environment and the rights of older people – should also be included within the Scottish human rights framework, thereby ‘demonstrat[ing] human rights leadership by securing adequate protection for all’ (2021: 6). It seems likely therefore that Scottish reform of capacity legislation will be carried out in a way that is mindful of the obligation to ‘respect, protect and fulfil human rights’ (Scott Review, 2022a: 33) and in a way that incorporates the values of the CRPD (Scott Review, 2022a: 4).

Though the consultation processes took place almost 20 years apart, many of the concerns expressed in Scotland regarding best interests in the 1990s were reiterated in the evidence that was submitted to the Oireachtas Joint Committee on Justice, Defence and Equality (the Joint Committee) tasked with considering the new Irish legislation. As mentioned previously, the Irish road to legislating in this area was not a short one. Between 2003 and 2006, the Law Reform Commission (Ireland) published two papers, the first called for the abolition of the wardship system,^16^ which was the legal mechanism in Ireland for making decisions on behalf of those who lack capacity and the second called for legislation to establish clear rules on when a person has the capacity to make healthcare decisions and the replacement of wardship with a guardianship system (Law Reform Commission (Ireland), 2003; Law Reform Commission, 2006). In the mid-2000s and early 2010s a series of Bills relating to decision-making and capacity were presented and debated in the upper and lower houses of the Oireachtas (Irish Parliament), however many of these legislative attempts lapsed due to the dissolution of the Seanad (upper house) and Dáil (lower house) in 2007 and 2011 respectively. Furthermore, the delay in legislating was attributed to the Irish financial crisis in the late 2000s, which led to the prioritisation of legislation required under the EU-IMF programme of financial support to Ireland in the early 2010s (Seanad Debate (Lynch), 2012).

In 2015, the Assisted Decision-Making (Capacity) Bill 2013 was eventually presented to both houses of the Oireachtas having undergone significant changes from the original Scheme issued in 2008. Critically, amongst those changes was the rejection of ‘best interests’ in favour of ‘will and preference’ as one of the guiding principles of the Bill. That shift is interesting for two reasons: first, it was a departure from the common law standard for decision-making on behalf of adults that lacked decision-making capacity, in contrast to what happened with the MCA. Secondly, the change was quite late in the consultation process, which highlighted the impact of advocacy and civil society groups on the legislative process, which is explored further below.

The applicability of the best interests test to decisions concerning persons lacking capacity in Ireland was established by *Re a Ward of Court (withholding medical treatment) (No 2)*^17^ and upheld as recently as *A v Hickey & Ors*.^18^ In the words of Lynch J in *Re a Ward of Court*:

I take the view that the proper and most satisfactory test to be applied by the Court in this case is the best interests test, ie, whether it is in the best interests of the ward that her life, such as it is at present, should be prolonged by the continuation of the abnormal artificial means of nourishment, or, whether she should be allowed to slip away naturally by the withdrawal of such abnormal artificial means(…)’^19^

Thus, as distinct from codifying the existing common law standard, the Irish legislature specifically legislated for a different standard, one which has been described as ‘a rights-based approach to decision making’ (Inclusion Ireland, 2022). This is worth noting because the Joint Committee received a number of submissions in support of the best interests standard as well as proposals to codify and clearly define it (Oireachtas Joint Committee, 2012a).^20^

It is entirely possible that the eagerness of the Irish legislature to comply with the CRPD and to abandon the best interests standard may stem from the explicit purpose of the legislation. It was acknowledged in the Explanatory Memorandum accompanying the Bill (Assisted Decision-Making (Capacity) Bill 2013: 2) and also during the course of the Joint Committee hearings and in the Dáil that the impetus for the new legislation was to enable Ireland to ratify the CRPD (Oireachtas Joint Committee (Stanton), 2012b: 25a; Dáil Debate, 2013).^21^ Furthermore, it was described as fulfilling the State’s requirement ‘to eliminate barriers preventing people with disabilities from enjoying their human rights and fundamental freedoms’ (Lynch, 2013), thereby emphasising the focus on the rights of persons with disabilities and human rights more broadly in the process. Consequently, it is possible that the Joint Committee chose to err on the side of caution by adopting ‘will and preferences’, which they were confident would be ‘CRPD compliant’, as opposed to codifying best interests, given the doubts that had been cast upon its suitability as the decision-making standard. For example, in the words of the Chair of the Joint Committee, David Stanton T.D.: ‘It would be terrible to pass legislation in the Houses and find we still cannot ratify the convention, which is one of the aims of the Bill.’ (Oireachtas Joint Committee (Stanton), 2012b: 25a). Furthermore, at consultation stage the Bill was described as ‘a proposed scheme which is up for grabs and can be changed, adapted and modified’ (Oireachtas Joint Committee (Stanton), 2012b: 26a), indicating the degree to which the Joint Committee was receptive to change.

Thus, as is the case in Scotland, human rights compliance played and continues to play a significant role in the Irish process. Arguably, however, the focus on human rights is less cohesive in Ireland, in comparison to a more unified approach in Scotland.^22^ Either way, it is apparent that having a clear end goal of reform that reflects the CRPD is likely to impact heavily on the direction of the legislation in the Irish and Scottish processes, in contrast to Northern Ireland, where CRPD compliance was not a specific objective of the MCA(NI) (Explanatory Notes MCA(NI) 2016: Part 1).

Legislating to give effect to the individual’s will and preferences appears to suggest, at least in principle, that Irish law is more focused on autonomy and self-determination, in line with the ethos of the CRPD. How matters operate in practice, however, remains to be seen. First, the attitude of the Irish courts to the related area of mental health law has, arguably, been quite paternalistic (Oireachtas Joint Committee (Carroll), 2012b: 11a; Oireachtas Joint Committee (Keys), 2012a: 24a; Craven, 2009; Kelly, 2017: Chapter 2). Given the potential for crossover between decision-making on behalf of adults that lack capacity and mental health law, there is scope for that paternalism to seep into mental capacity law. Secondly, amongst the guiding principles (of which will and preferences is one) is the requirement that intervention benefit the individual (ADM(C)A, s 8(7)(e)), and it is unclear how potential conflict between these two principles will be resolved.

Thirdly, it is worth commenting on the significance and impact of the wording and language used, whether it is ‘best interests’, ‘benefit’ or ‘will and preferences’. It could be argued that the precise nomenclature may not be as important as it first appears and that what really makes a difference is how the term is implemented on the ground. Or to put it another way; legislation is not always interpreted in a way that its drafters would have expected or hoped. For example, Ruck Keene and Ward aptly demonstrate this in their account of the ‘journey [in Scotland] away from (…) giving primacy to the “will and preferences” of the adult or their best interpretation, towards a “best interests” approach in the sense used, and criticised, by the UN Committee’ (Ruck Keene and Ward, 2016: 35). They contrast this with the case law in England & Wales, which exemplifies ‘a trend towards paying greater heed to the individual’s wishes and feelings’ despite the term ‘best interests’ (Ruck Keene and Ward, 2016: 36). Furthermore, they discuss a series of cases in Scotland where the term best interests was used, despite the primacy of the benefit principle in the AWIA (Ruck Keene and Ward, 2016). They make clear the importance of how a term is understood and applied by the courts, as well as the health and welfare practitioners who use it on a daily basis. Thus, the language used in legislation must be viewed in context and in light of the meaning that it is given by those who are tasked with implementing it.

Nevertheless, Donnelly has convincingly argued for a change in terminology from best interests, albeit recognising that challenges associated with the term cannot be addressed by a simple change in terminology to will and preferences (2016: 331). She argues that best interests now signifies ‘something quite different to its original meaning’, as noted above. She maintains that the ‘dissonance between the original, objective, meaning of the term and its current meaning under the MCA’ creates difficulties where the term is retained but with the intention that it will mean something else (what she describes as serving as ‘a shorthand for something different’) (2016: 331). This, she argues, is because ‘decision-makers’ intuitions associated with the old meaning will continue to assert themselves with the result that the underlying conceptual shift cannot be delivered upon’ (2016: 331). This seems entirely plausible when one considers the original meaning of ‘best interests’ in *Re F* as an objective and medically-focused standard and perhaps accounts for some of the difficulty in the implementation of the MCA (House of Lords, 2014). Thus, we are left with the need to revisit the issue after time has passed in order to assess the degree to which implementation of legislation has reflected the human rights norms and best practice that they strove to uphold.

### 4.2 Human rights in Northern Ireland

The impact of human rights is also evident when we consider the legislation in Northern Ireland, albeit with a different outcome in relation to the decision-making standard. The ‘fusion’ approach adopted by the Northern Irish legislature is clearly reflective of international human rights best practice. The bringing together of mental health and capacity law in one statute with best interests and impaired decision-making capacity as the criteria, irrespective of whether the intervention relates to physical or mental health,^23^ also reflects the core values of the CRPD. Furthermore, given that mental health law is openly and clearly based on a form of disability, it could be argued that replacing it with a fusion approach represents a more significant move towards equal recognition before the law than choosing one term over another.^24^ Furthermore, the fusion approach also reflects much of the ethos of the Bamford Review established in 2002 to consider mental health law and the law relating to individuals with a learning disability. The Bamford Review sought to reduce the stigma associated with mental health issues on the basis that having ‘one law for decisions about physical illness and another for mental illness’ is an ‘anomalous, confusing and unjust’ position (Bamford Review, 2007: para. 4.64; see also Davidson et al., 2016). Consequently, its proposals effectively underpinned much of the MCA(NI).

The assimilation of decision-making for mental and physical health is welcome given that there have been calls for some time for a fusion law, which would reduce stigma and the potential for discrimination against those with mental health issues (Dawson and Szmukler, 2006; O'Brien, 2010; Szmukler et al., 2014). Indeed, the Scott Review said that the case for fusion has recently become clearer given the potential for confusion that is generated by multiple legal frameworks – mental health, capacity and adult protection – (2022a) and that ‘the ultimate long-term goal should be one of fused mental health and capacity legislation’ (2022b: 93). The desirability of a fusion approach in the future has also been acknowledged in Ireland (Expert Group on the Review of the Mental Health Act 2001, 2014: 11) and debated in England & Wales (Independent Review of the Mental Health Act 1983, 2018: 222-7). Thus, while one might be forgiven for querying if the rights of the individual, particularly the individual with a disability, were at the forefront of the minds of those drafting the MCA(NI) given the retention of best interests, the MCA(NI) is nevertheless a ground-breaking piece of legislation and a considerable step forward in challenging discrimination on the basis of mental health.

### 4.3 Disagreement on the CRPD: The effect in Northern Ireland, Scotland and Ireland

Where Northern Ireland diverges from Scotland and Ireland is related to the effect that international disagreement on the correctness of the CRPD Committee’s interpretation had on the final legislation. The compliance of best interests and the proposed Northern Irish legislation with the CRPD was discussed in much of the evidence submitted to the Ad Hoc Joint Committee formed to consider the Bill during the consultation phase (McSherry, 2015; mencap, 2015; Disability Action, 2015; Ruck Keene, 2015; Essex Autonomy Project Martin, 2015; Law Centre (NI), 2015). Despite this and though disagreement regarding the CRPD was also a feature of the oral hearings, the Ad Hoc Joint Committee did not dedicate significant space in its final report to discussing CRPD compliance. Instead, the Committee simply summarised the debate about best interests and the CRPD, quoted the concern of departmental officials^25^ about the contested status of the CRPD Committee’s interpretation and accepted the Department had ‘attempted to take account of emerging thinking in relation to the UNCRPD’ in the Bill (Ad Hoc Joint Committee, 2016: 13). The Committee found that DHSSPS had ‘attempted to achieve an appropriate balance’ between ‘the implications of the UNCRPD and the ECHR in terms of the concept of best interests’ (Ad Hoc Joint Committee, 2016: 17). This approach seems to be consistent with the views of a number of commentators; Harper et al argue that ‘[i]t is only on the basis of a debatable interpretation of the UNCRPD (…) that the Northern Ireland Act seems incompatible’ (Harper, Davidson, and McClelland, 2016: 66). Moreover, there are questions as to whether any domestic assisted decision-making framework has complied fully with the CRPD Committee’s interpretation of Article 12, or if any such framework would ever be capable of such compliance.

Among the submissions received on the relationship between the Mental Capacity Bill (Northern Ireland) and the CRPD was evidence from the Northern Ireland Human Rights Commission (NIHRC), the non-departmental public body responsible for ensuring that government and public authorities protect, respect and fulfil human rights in Northern Ireland. The NIHRC discussed the relationship between the CRPD and best interests but stopped well short of suggesting it should be removed from the Bill (NIHRC, 2015). Instead, the NIHRC advised that consideration ought to be given to whether the legislation could further reflect the CRPD by ‘using the language of Article 12 and replacing “past and present wishes and feelings” with the terms “will and preferences”’ (NIHRC, 2015: 3). Thus, it advocated for will and preferences to be a factor within an assessment of best interests, rather than to replace the concept entirely. Furthermore, the NIHRC stated at the outset that it acknowledged ‘a number of disparities and contradictions in the standards set down by the ECt.HR and the standard set by the UNCRPD Committee’ (NIHRC, 2015: 3), for example in relation to deprivation of liberty and medical treatment without consent (NIHRC, 2015: 10). This concern regarding the relationship between the CRPD, as interpreted by the CRPD Committee, and the ECHR was shared by the relevant government departments in Northern Ireland (i.e. DHSSPS and DOJ) and was reflected in the final report of the Ad Hoc Committee (2016: 13). Consequently, it could be argued that the Northern Irish legislature was influenced more by the ECHR in the context of capacity law than the CRPD, perhaps reflecting some of the concerns outlined above about the coherence of the CRPD with other human rights instruments.

Whilst the CRPD has been prominent in discussions in Scotland, the Scott Review was also cognisant of the international disagreement about Article 12. It was noted that since the publication of the CRPD Committee’s General Comment, ‘there has been extensive debate across the world about whether the Committee’s conclusions go further than the Convention requires, and about tensions between that Convention, the European Convention on Human Rights, and other international human rights treaties’ (Scott Review, 2022a: 90). Critically:

We understand that Scotland, as part of the UK, has committed to reform its law towards compliance with the UNCRPD – the Convention – and *to take account* of direction from the United Nations Committee on the Rights of Persons with Disabilities on how to do this (2022a: 90 emphasis added).

Arguably, this indicates a view in Scotland that compliance with the CRPD Committee’s General Comment must be tempered and balanced against responsibilities and rights that may arise by virtue of other human rights instruments, as outlined above.^26^ Thus, while many core values of the CRPD are clearly prominent in the recommendations of the Scott Review, the membership of the Review ‘have not been persuaded that an absolutist approach is as yet possible or even necessarily desirable’ (2022b: 27).

Curiously absent from the consultation on the Irish legislation was the widespread international disagreement about Article 12, despite some concern during the Joint Committee debates about such compliance (Oireachtas Joint Committee (Stanton), 2012b: 25a).^27^ Best interests was thought to be appropriate in some quarters,^28^ however the Joint Committee concluded that an alternative will and preferences model was preferable, as it was thought to better promote the principles of autonomy and patient empowerment, in line with the CRPD. Unlike Northern Ireland, there appeared to be no voices within the Irish process openly challenging the need for will and preferences nor the correctness of the interpretation of the CRPD Committee. As will be discussed in the next section, this strong, vocal and consistent messaging within the Irish process was not accidental, but largely by design.

### 4.4 The influence of external actors on the legislative process in Ireland, Scotland and Northern Ireland

This article now turns to look at the way in which the shift away from best interests in Ireland occurred. Throughout the bulk of its progression, the Irish draft legislation had referred to ‘best interests’ (Scheme of Mental Capacity Bill 2008, Head 3), to be determined in an almost identical fashion to best interests under the MCA. For example, similar to section 4(6) of the MCA, Head 3 1(i) of the original Bill required consideration of the person’s past and present wishes and feelings, beliefs and values and any other factors that would likely be considered by the person. It was quite late in the day, so to speak, that this was changed, following what appeared to be very strong and vocal opposition. This came from a civil society coalition co-led by Amnesty Ireland and the Centre for Disability Law and Policy based at National University of Ireland, Galway (CDLP), as well as a number of related interest groups, such as Inclusion Ireland and it led to the removal of best interests from the Bill, amongst other changes.^29^ This opposition to best interests was also supported by the Irish Human Rights Commission (Oireachtas Joint Committee (Manning and Hogan), 2012b) and reflected in the evidence given by the representative from Office of the Council of Europe Commissioner for Human Rights (Oireachtas Joint Committee (Sivonen), 2012b). Amid other criticisms of best interests expressed to the Joint Committee in evidence given by these groups and individuals was its inappropriateness to adult capacity as a child welfare concept and its inherent potential for paternalism.^30^ This view of best interests was clearly reflected in the final report of the Joint Committee:

Concerns were raised by the use of the best interests model. It was stated to the Committee that this is a model of the past. It demonstrates the paternalistic view of trying to determine what the best interests of a person are (…) It was put to the Committee that best interests are usually what the professionals see as the person’s best interests.

Its inclusion in the legislation was questioned and it was suggested that it was contrary to the UN Convention on the Rights of People [sic] with Disabilities, which never mentions best interests in relation to adults (Oireachtas Joint Committee, 2012a: Background).

This section of the report draws directly on the evidence submitted by individuals, the civil society coalition and interest groups. For example, the ‘model of the past’ comment comes almost verbatim from the evidence given by the Office of the Council of Europe Commissioner for Human Rights and is also present in the evidence given by Inclusion Ireland and the CDLP. References to best interests being contrary to the CRPD can be traced back to oral and/or written evidence given by both groups (Oireachtas Joint Committee (Carroll), 2012b: 11a; CDLP, 2012: 6). They also expressly referred to the inappropriateness of a child law concept being applied to adults, to the risk of paternalism and the view that best interests is usually what the professional views as the best interests of the individual (Oireachtas Joint Committee (Carroll), 2012b: 11a; CDLP, 2012: 21).

Similar arguments against best interests provided some of the basis for its rejection in Scotland in the 1990s (Scottish Law Commission, 1995: para. 2.50). This aspect of the Irish processes in itself is noteworthy; by the time the Joint Oireachtas Committee was in receipt of these submissions, it is fair to say that best interests in England and Wales had moved on significantly to encompass a range of medical and non-medical factors. Yet, many of the same arguments against it were advanced during the reform processes in Scotland and Ireland. In any event, the change in standard appears to highlight the degree to which civil society and advocacy groups were able to influence the legislature during the reform process. This is not unique to the Irish context; for example, recent dialogue about legal reform in Scotland also highlights how external special interest groups are directly involved in legal reform. The Scott Review team actively engaged with service users, civil society and advocacy groups and practitioners, consistent with its approach in earlier human rights consultations.^31^ Furthermore, reflecting on the legislative process for the AWIA, the Scottish Law Commission explicitly noted submissions from groups such as the Lothian Home for Mentally Disabled People, which argued that best interests was ‘too paternalistic’ (Scottish Law Commission, 1995: para. 2.50). While the inclusion of these voices may not yield the same outcome in every jurisdiction, it indicates that their views are being listened to and considered, to varying degrees, within these ‘law-making’ processes.^32^

The participation of civil society and advocacy groups in Northern Ireland is also evident and this extensive engagement was argued to have ‘[built] on the inclusive approach taken in the Bamford Review’ (Harper et al., 2016: 60). The Ad Hoc Committee tasked with reviewing the Mental Capacity Bill in Northern Ireland received over 50 written submissions from individuals, professionals, advocacy groups, academics and public bodies. Even before the Ad Hoc Committee review of the Mental Capacity Bill, however, both the departmental consultation between the Department of Health, Social Service and Public Safety (‘DHSSPS’) and Department of Justice (‘DOJ’) and the Bamford Review itself had wide consultative approaches.^33^ It has been argued by Harper et al that the wide consultation process resulted in the government committing to a fusion approach to the law, as opposed to the development of separate capacity legislation alongside a mental health framework, as was originally desired by the respective departments (Harper et al., 2016: 60; DHSSPS and DOJ, 2015: 4; 13-4). What the wide consultation did not do in Northern Ireland, however, was persuade the legislature that it was either necessary or desirable for best interests to be abandoned in favour of will and preferences.

This differing outcome may be due to a number of factors. First, the purpose of the Irish legislation was to enable Ireland to ratify the CRPD. By contrast, the purpose of the MCA(NI) was to bring Northern Ireland into line with the rest of the UK – which had specific capacity legislation for some time – and to reform the Mental Health Order as it was ‘out of step with the growing recognition of the right to personal autonomy’ (Explanatory Notes MCA(NI) 2016: para. 5).^34^ Secondly, and of greater significance, is the balance of evidence given to the Joint Committee in each jurisdiction and the approaches of each Joint Committee to the legislative consultations. The Northern Irish Joint Committee was essentially satisfied with the status quo in the absence of persuasive evidence in favour of an alternative. While the process saw extensive submissions, considerable disagreement regarding the relationship between the CRPD and best interests was reflected strongly in the evidence given. Furthermore, not only was best interests the consistent position advanced by the relevant government departments throughout the earlier policy and consultation stages (DHSSPS and DOJ, 2015) but it is also a reflection of the Bamford Review, which had explained one of its core values – ‘benefit’ – in terms of ‘best interests’ (Bamford Review, 2007: 38; Law Centre (NI), 2014:9). The current Scottish reform started from a different position, recognising that the status quo is not satisfactory and law reform is needed for Scottish law to comply with the CRPD and for Scotland to achieve its aim of being a human rights leader internationally. Similarly, the Irish draft legislation was malleable from the start, categorised as changeable, adaptable and modifiable. Specifically, the Chair of the Joint Committee remarked on the purpose of the evidentiary hearings as follows:

This is why we are having these hearing. This is a complex Bill and impacts on many people and families. Their lives can be improved if we get this right. It is hoped the system will work. The delegations should feel free to keep in contact with the committee as the Bill progresses (…) (Oireachtas Joint Committee (Stanton), 2012b: 26a)

When one considers the Irish and Northern Irish consultation processes, the balance of the evidence appears to prove critical. The Irish consultation process had considerable presence from Irish academics and interest groups who made persuasive written and oral submissions regarding the need for a will and preferences model. Absent from this debate was the correctness or otherwise of the CRPD Committee’s General Comment. Instead, it was taken as given by the Joint Committee that will and preferences was necessary in order to achieve CRPD compliance. By contrast, the consultation in Northern Ireland engaged with academics and practitioners from both Ireland and the United Kingdom, thereby resulting in multiple competing voices as to the interpretation of the CRPD and necessity for will and preferences.^35^ The consistency of message in Ireland was not by chance – it was a deliberate strategy by a civil society coalition all advocating strongly for a will and preferences model. This strategy element is reinforced by the reflections of an active member of that coalition, Professor Eilionóir Flynn, on the passing of the ADM(C)A:

The coalition decided to develop a ‘Principles’ document that we hoped would influence the eventual capacity legislation. During the development of the principles, some of the groups involved in the coalition were invited to present views (…) as part of the pre-legislative scrutiny process (…) We had hoped to have agreed the principles by then, but (…) it was not possible to get sign-off from all the key players in time for the Committee hearings. Instead, efforts to align our positions were made as much as possible among those of us presenting to the Committee (…)’ (Flynn, 2021b: 106)

The consistent messaging strategy was supplemented by a series of public conferences and workshops in 2011 and 2012, with the aim of ‘sending a specific political message’ – namely that ‘supported decision-making was both necessary and feasible and could be recognised in the Irish legislative context’ (Flynn, 2022: 125). Arguably, ‘normalising’ a particular practice in this way is a powerful method of inciting legal change and has already been successful in other contexts, for example, in relation to contraceptive sterilisation (Lewis, 2012).

There was no evidence of a unified persuasive voice or clear strategy opposed to best interests during the Northern Irish process and it may not have been necessary in the current Scottish reform process as the language of will and preferences was already being used in the context of mental capacity.

## 5 Conclusion

This article has explored the relevant legislative (reform) processes in the area of assisted decision-making in Scotland, Ireland and Northern Ireland, focusing on what led and continues to lead each jurisdiction to adopt particular legal decision-making standards. One could argue, as we have done, that there is more of an appetite in Scotland and Ireland to align the relevant decision-making standard with the CRPD than in Northern Ireland. Arguably, the current focus on human rights leadership in Scotland may have partly driven the prominence that is being given to the values of the CRPD in recent reform debates, though there is clearly still doubt about the CRPD Committee’s General Comment. Conversely, the CRPD could not be ratified until Ireland legislated in the area of decision-making capacity. Arguably, these factors are likely to have driven the law in specific directions, particularly when combined with strong, vocal and critically strategic representation from Irish civil society groups.

Civil society and advocacy groups have played a role, to varying degrees, in the ‘law-making’ processes in all jurisdictions. While they were heavily involved in the process in Northern Ireland – seemingly ultimately leading to the creation of a fusion law – the legislature was unconvinced by claims that best interests needed to be rejected in favour of a will and preferences model. This can perhaps partly be attributed to the degree to which best interests represented the position of the Bamford Review and the relevant Northern Irish government departments. However, equally so, it is also a reflection of the considerable international debate as to the CRPD Committee’s General Comment, which is supported by some of the evidence presented to the Joint Committee. This disagreement, coupled with a far stronger presence of UK professionals – both academics and practitioners from England & Wales – in the Northern Irish process, may have led to those voices getting lost in a way that did not happen in Ireland, where there was a clear strategy employed by those advocating for a will and preferences model. Furthermore, the heavy presence of UK based professionals may also have supported a starting point of best interests being regarded as ‘the norm’ rather than an exception. In any event, this explicit acceptance of best interests over will and preferences may leave the Northern Irish framework open to criticism for failing to embrace a key aspect of the CRPD.

Despite the move to will and preferences, concerns also persist in relation to the Irish framework, not least because of the requirement that intervention should benefit the individual. While the guiding principle of benefit may be well-meaning, it raises questions as to compliance with the CRPD and has been the subject of criticism (Donnelly, 2016: 331). For example, if the requirement to benefit the individual contained in the ADM(C)A is interpreted as medical benefit or in another way that results in a ‘detrimental act’ (representing the will and preferences of the individual) being superseded, then legitimate questions may be asked regarding any difference, in practice, between the Irish approach and the ‘best interests’ basis for decision-making.^36^ Looking to Scotland as a jurisdiction which has utilised the concept of benefit or over 20 years, is not helpful either, as Ruck Keene and Ward note:

[T]here have been no equivalent progressive and differing lines of development [in Scotland] in relation to whether (…) the benefit principle equates to a best interests test, or the relative weight to be given to the benefit principle when balanced against the others, particularly the past and present wishes and feelings of the adult (Ruck Keene and Ward, 2016: 31).

In any event, whether unintentional or deliberate on the part of the Irish legislature, a guiding principle of benefit leaves the door open for paternalism to continue to influence this area of law, leading to questions about adherence to the CRPD – the very Treaty to which the legislation was supposed to give effect. This is certainly an aspect of the Irish legislation that warrants further scrutiny, though a thorough analysis is beyond the scope of this article. In any event, what this and some of the other aspects of the domestic frameworks discussed above highlight is the fact that no domestic legislation in the UK and Ireland is completely ‘CRPD-compliant’.

The move towards a social model of disability and fully realising and protecting the rights of people with disabilities in the CRPD has led to calls for an approach based on will and preferences. In principle, such an approach is intended to maximise the voice of the individual and place them at the centre of decision-making. With that said, it is difficult to predict what impact the differing concepts of decision-making will have in practice given the relative recency and commencement status of the legislation in Ireland and Northern Ireland. While the decision-making standard adopted by the legislature is undoubtedly important, arguably how the legislation is interpreted and implemented in practice will be most critical in determining the extent to which the reforms maximise the rights of the individual.

## Footnotes

1 This work was supported by the ‘Balancing Best Interests in Health Care, Ethics and Law (BABEL)’ Collaborative Award from the 10.13039/100010269Wellcome Trust [209841/Z/17/Z] It is worth briefly setting out some terminology that may prove confusing if not addressed at this stage. Traditionally ‘substitute judgment’ (or ‘substitute decision-making’) was the term used to describe a decision on behalf of a person lacking capacity which reflected what the individual would have chosen themselves if they had capacity. It was favoured by some parts of the United States (see *Re Quinlan* 70 NJ 10 (1976); *Re AC* 573 A 2d 1235 (DC 1990)). More recently, substitute decision-making has come to mean any decision on behalf of a person who lacks capacity, whether informed by best interests or another standard. For the duration of this piece ‘substitute decision-making’ can be understand as the latter.

2 It is worth stating at the outset that this piece confines itself to considering the relevant capacity legislation in the UK jurisdictions and Ireland and though cognizant of the potential implications from a CRPD perspective, does not consider the relevant mental health legislation.

3 For example, see *NHS Trust A v M; NHS Trust B v H* [2001] 2 WLR 942, which was the first case to consider withdrawal of artificial hydration and nutrition from patients in a persistent vegetative state post the Human Rights Act 1998 coming into force.

4 There are differing opinions as to whether the CRPD and treaties like it are an example of hard law or soft law. This discrepancy appears to relate to the understanding of ‘hard’ and ‘soft’ in the context of international law. The CRPD is ‘hard law’ in the sense that it is a treaty signed by the United Kingdom and Ireland. As noted by the Joint Committee on Human Rights (UK): ‘Treaties (…) are legally binding on the state in international law. A violation of a treaty obligation is an internationally wrongful act which has serious consequences for the State in international law’ (Joint Committee on Human Rights, 2012: 13-14). This distinguishes the CRPD from soft law mechanisms frequently used by the UN such as Resolutions of the General Assembly. However, ‘hard’ versus ‘soft’ can be used to distinguish other kinds of legal situations; for example, it is sometimes used to describe a treaty that is not directly enforceable within a jurisdiction because the system is dualist (like the UK) and hasn’t yet incorporated the treaty through domestic legislation. Furthermore, it has been used to contrast instruments that refer to ‘precise wording’ and ‘exact obligations’ (hard) with instruments that are comprised of ‘norms’ or ‘general goals’ (i.e. soft) (Chinkin, 1989).

5 [2017] ECHR 273.

6 *Re F: (An Adult: Sterilisation)* [1990] 2 AC 1; As stated by Lord Brandon, ‘the lawfulness of a doctor operating on (…) an adult patient disabled from giving consent will depend (…) on the question whether the operation (…) is in the best interests of the patient concerned’. See also *Airedale NHS Trust v Bland* [1993] A.C. 789.

7 *Bolam v. Friern Hospital Management Committee* [1957] 1 W.L.R. 582.

8 *Re Y (Mental Patient: Bone Marrow)* [1997] Fam. 110, para 116.

9 *An NHS Trust v X* [2005] EWCA Civ 1145.

10 *Aintree University Hospitals NHS Trust v James* [2013] COPLR 492, para 502.

11 See *Aintree University Hospitals NHS Foundation Trust v James* [2013] UKSC 67; *Sheffield Teaching Hospitals NHS Foundation Trust v TH* [2014] EWCOP 4; *Re D (Withdrawal of Treatment)* [2012] EWCOP 885; *Barnsley Hospitals NHS Foundation Trust v MSP* [2020] EWCOP 26

12 [1997] 2 FLR 426.

13 *Re F* [1990] 2 A.C. 1, para. 18; *Re MB (An Adult: Medical Treatment)* [1997] 2 FLR 426, para. 439.

14 See also the National Taskforce for Human Rights, ‘Leadership Report’ (2021) Chapter 2 and the terms of reference of the Scott Review, which include ‘making recommendations that gives effect to the rights, will and preferences of the individual by ensuring that mental health, incapacity and adult support and protection legislation reflects people’s social, economic and cultural rights including UNCRPD and ECHR requirements’ (2020).

15 It is worth noting that in its investigation, the UN Committee on the Rights of Persons with Disabilities commented favourably at p. 1 on a number of legislative and policy measures in Scotland – including ‘A Fairer Scotland for Disabled People’ – which had been drafted / implemented with input from persons with disabilities.

16 Wardship ‘originated in the Crown prerogative for the purpose of acting as guardian of people with legal disabilities’ in other words, *parens patriae*. While there is some debate as to whether *parens patriae* survived Irish independence from the United Kingdom and the constitutional changes in Ireland in 1922, it remains the case that wardship was the mechanism by which decisions are made for adults found to lack decision-making capacity at the time of the Law Reform Commission’s paper [84-87]. This will remain the case until it is replaced by the ADM(C)A, which is yet to be commenced.

17 *Re a Ward of Court (withholding medical treatment) (No 2)* [1996] 2 IR 79

18 [2021] IEHC 318

19 [1996] 2 IR 79, p. 98. This approach was upheld by the Supreme Court with Hamilton CJ stating: ‘In the exercise of this jurisdiction [over wards of court] the court's prime and paramount consideration must be the best interests of the ward [p. 106].’

20 See for example, the submission from the Mental Health Commission on the proposed Bill (p. 9): ‘The overtones of paternalism in use in a test of best interests are now balanced in Head 3 with the focus on the person at the centre of the decision or action. The section mandates a range of considerations in arriving at a decision on what is in the best interests of the person.’ See also paragraph 2.3.2 of the submission from the Alzheimer Society of Ireland: ‘We also welcome the clear definition of best interests, an area which has to date been problematic in practice and prone to a wide range of interpretation. The imperative to take into account present and past wishes where known, and to consult with the family and friends of the incapacitated person is particularly welcome.’ Submissions are available in Appendix 4 of the Report of the Joint Committee.

21 See also the Programme for Government 2011-2016, wherein it was stated that the government would ‘reform the law on mental capacity to ensure the greatest degree of autonomy for people with intellectual disabilities or suffering with mental illnesses in line with the UN Convention on the Rights of Persons with Disabilities’ (Government of Ireland, 2011: 55)

22 One could assert this for a few reasons; first, the reports from both the First Minister’s Advisory Group on Human Rights Leadership in 2018 and the Human Rights Taskforce in 2021 were wide-ranging, encompassing numerous areas in need of reform and improvement in order for Scotland to be a world leader in human rights. These reports underpin a number of (proposed) reforms in Scotland, thereby leading to coherence and focus on human rights, rather than an ad hoc inclusion of human rights in reform processes (First Minister’s Advisory Group on Human Rights Leadership, 2018; National Taskforce for Human Rights, 2021. Responsibility for Irish human rights strategy rests with the Irish Human Rights and Equality Commission, which is also the mechanism for a national independent monitoring framework for the CRPD in Ireland. It is a relatively new organization established under the Irish Human Rights and Equality Commission Act 2014. It replaced the Irish Human Rights Commission, which was disbanded in 2014. While the IHREC has has released a three strategy statements (2016-8; 2019-21; 2022-25), all of which refer to the CRPD, the detail regarding how these aims will be achieved is quite scant. Thus, in contrast to Scotland, the link between strategy and implementation is not completely clear in Ireland. Second, the courts in Ireland have had a role for quite some time in recognising rights in Ireland on what could be thought of as an ad hoc basis. Ireland has had a written constitution since the foundation of the State meaning that rights in Ireland are enumerated i.e. contained in the Bunreacht na hÉireann (the Irish Constitution) and unenumerated. This means that the courts have had a role in interpreting the Constitution as protecting certain unwritten rights, for example, the right to bodily integrity as established in *Ryan v Attorney General* [1965] IR 294 or marital privacy as established in *McGee v Attorney General* [1974] IR 284.

23 This is distinct from the more ‘traditional’ criteria of presence of a mental disorder and risk to themselves or others.

24 Sincere thanks to an anonymous reviewer for this observation.

25 Department of Health, Social Services and Public Safety (DHSSPS) and Department of Justice (DOJ) to the Ad Hoc Committee.

26 It could also be argued that a similar approach to balance is also evident in the comments contained in the Consultation concerning retention of non-consensual treatment (Scott Review, 2022a: 45). The necessity of non-consensual treatment in certain circumstances is a view also shared in some form or other by other commentators (Szmukler et al., 2014: 247; Davidson et al., 2016: 31).

27 Furthermore, in giving the background to the Bill, the Committee opens with: ‘Ireland was one of the first countries to sign the UN Convention on the Rights of Persons with Disabilities but as yet, is not in a position to ratify it.’ Arguably, this signifies that the CRPD was at the forefront of the minds of the Committee members when considering the proposed legislation. This may also explain why the ADM(C)A does not have a requirement that the person be suffering from ‘an impairment’ or ‘disturbance’ of the mind or brain in order to lack capacity, in other words there is no requirement for the individual to have a disorder, disability or diagnosis of some kind. This requirement has been argued to be potentially ‘unnecessary and discriminatory’ by Davidson et al. (2016: 39).

28 See footnote 20.

29 Civil society groups were also credited with the change of the title of the Bill from the Mental Capacity Bill to the Assisted Decision-Making (Capacity) Act to better reflect ‘the fact that, rather than merely introducing new tools for assessing capacity (…) the primary focus of the 2015 Act is on supporting a person as far as possible to exercise their decision-making autonomy’ (Flynn, 2021a: Foreword). Furthermore, the Decision Support Service, which will be responsible for much of the functioning of the Act, was going to be called the Office of Public Guardian until the ‘connotations of paternalism contrary to the ethos and purpose of the Act’ were highlighted (Flynn, 2021a: Foreword).

30 See the submission from the National Federation of Voluntary Bodies Providing Services to People with Intellectual Disability p. 5: ‘The best interests principle emerged from the children’s perspective and it is increasingly considered inappropriate in relation to adults.’ Submissions are available in Appendix 4 of the Report of the Joint Committee.

31 The Executive of the Scottish Mental Health Law Review (Scott Review) has a membership of 5 people, 2 of which are individuals with first hand experiences of mental health services and the effect of capacity legislation.

32 For example, it is stated in the Terms of Reference of the Scott Review that ‘the review will ensure that persons with lived experience are at the centre of the review and fully involved in making recommendations for the final report’ (Scottish Mental Health Law Review, 2020). Furthermore, amongst the system wide changes being advised by the Scott Review is ‘[s]ignificant lived experience input at all levels of service delivery, and in the development of law, policy and practice’, again highlighting the importance that is being places on these views in Scotland (2022a: 41). The Consultation document also explicitly states that the proposals for change have been ‘developed in partnership with people with lived experience, including unpaid carers, and mental health practitioners through a series of Advisory Groups and Reference Groups’ (2022a: 1). Finally, the Final Report states: ‘Lived experience and human rights are at the heart of the Scottish Mental Health Law Review (…) In line with human rights thinking and principles, the voices of lived experience – of people – have been represented throughout every aspect of our work…’ (Scottish Mental Health Law Review, 2020b: 22)

33 For example, the Bamford Review was a culmination of ten Expert Working Committees and had input from professionals from Great Britain, the Republic of Ireland, New Zealand, Canada and the Netherlands in forming its proposals (Bamford Review (2007): i–iii; Annex 2).

34 See Explanatory Notes for the Mental Capacity Act (Northern Ireland) 2016. The introductory text of the Act also refers to making provision in connection with the Convention on the International Protection of Adults.

35 See the debate between Dr Eilionóir Flynn (as she was then) of the Centre for Disability Law and Policy (NUIG) and Dr Colin Harper of the Law Centre (NI) regarding the necessity of legislating for will and preferences to comply with the CRPD (Ad Hoc Joint Committee, 2015: 5–10). Furthermore, for example, all of the academics and practitioners who attended the roundtable for international experts and academics work(ed) for universities or practice(d) in the United Kingdom (and more specifically England & Wales).

36 The ADM(C)A also requires that due regard be given to ‘the need to respect the right of the relevant person to dignity, bodily integrity, privacy, autonomy’ in respect of interventions (ADM(C)A 2015 s 8(6)(b)), so it is possible that perceived benefit may be understood as synonymous with respecting the will and preferences of the individual.
